# ﻿Copulatory mechanism and genital coupling of the longhorn beetle *Moechotypadiphysis* (Coleoptera, Cerambycidae)

**DOI:** 10.3897/zookeys.1234.140491

**Published:** 2025-04-17

**Authors:** Dan-Wen Long, Xin Tong

**Affiliations:** 1 Guangxi Key Laboratory of Agro-environment and Agric-products Safety, National Demonstration Center for Experimental Plant Science Education, College of Agriculture, Guangxi University, Nanning 530004, Guangxi, China Guangxi University Nanning China

**Keywords:** Copulation, copulatory mechanism, endophallus, functional morphology, genital structure, insemination, mating behaviour

## Abstract

The function of insect external genitalia has played a significant role in exploring insect mating mechanisms and male fertilization strategies. However, due to the privacy of genital coupling, insect copulatory mechanisms have only been investigated in a few insect groups. In this study, we observed the mating behavior using freeze-fixated pairs in copula to reveal the copulatory mechanism of the longhorn beetle *Moechotypadiphysis* (Pascoe, 1871). At the beginning stage of mating, the male *M.diphysis* usually takes 30 min to control the female and then extends its median lobe and endophallus. Approximately 80% of males (19/24) of *M.diphysis* exhibit multiple expansions (the membranous endophallus expands and enters into the female genital tract), ranging from two to five times. There are two types of expansions: short ones lasting for 1.4 to 49 s and long ones ranging from 1.03 to 7.23 min. During copulation, male tarsi continuously grasped the female elytra, thorax, and abdomen to help the male to initiate and maintain copulation. Male genital structures are closely connected to female genital structures: the apical phallomere and flagellum on the male endophallus contacting the bursa copulatrix duct and the spermathecal duct of the female, and the abundant microstructures on the surface of the everted male endophallus directly anchoring the female genital tract. Finally, we discuss the possible reasons for the evolution of their complex mating-related structures. Our research will help to explore the evolutionary mechanisms of insect genital structures.

## ﻿Introduction

Firm coupling of male and female genitalia during copulation is critical to the success of sperm transfer for insects (Stutt and Siva-Jothy 2001; Chapman 2013; Matthews and Matthews 2010). Therefore, in order to guarantee the stability of genital coupling, males usually evolve diverse reproductive and non-reproductive structures to serve to manipulate and stimulate females, such as claws, hooks, and spines (Arnqvist and Nilsson 2000; Arnqvist and Rowe 2002a, b, 2005; Chapman et al. 2003; Wilson and Cranston 2010, 2014; Simmons 2014; Woller and Song 2017; Wulff et al. 2017). Understanding the roles of mating-related structures in copulation is important for revealing the functional morphology of insect genitalia (Wulff et al. 2017; Kelly and Moore 2016; Woller and Song 2017; Tong and Hua 2019). However, due to the difficulty of observing internal genital structures during copulation, relevant research is very limited.

In recent years, an effective method of freeze-fixation of copulating pairs has been proposed to examine the internal coupling of male and female genitalia (Grieshop and Polak 2012; Dougherty et al. 2015; Zhong et al. 2015; Tong et al. 2017; Woller and Song 2017; Lyu et al. 2018). However, this method has not been applied to the vast majority of insects, including Cerambycidae.

Many studies have delved into the structural functions associated with the mating behavior of longhorn beetles (Galford 1977; Hughes 1981; Akutsu and Kuboki 1983a, b; Iwabuchi 1985; Kuboki et al. 1985; Bianchi et al. 1988; Wang et al. 1990, 1991). Notably, Hubweber and Schmitt (2010) have explored the morphological diversity and functions of specific external genital structures within Cerambycidae. Bi et al. (2022, 2024) have also reported the enormous diversity of male endophallus in morphological structures. However, due to technological limitations, the examination of how the internal genital structures of male and female longhorn beetles interacted during mating remains limited. The application of freeze-fixated technology may gain insights into the internal coupling of male and female genitalia and unravel the functional morphology of Cerambycidae.

*Moechotypadiphysis* (Pascoe, 1871) (Cerambycidae, Lamiinae, Crossotini) is a common species in China, Russia, Japan and Korea, where it has been identified as an economically significant pest species. Investigating the mating behavior and the genital coupling of *M.diphysis* is crucial for understanding the functional morphology of the genital structures of Cerambycidae.

In this study, we observed the mating process of *M.diphysis* by dissecting the male and female genital systems in genital copula to analyze the functional morphology of male and female genital structures, to shed light on their mating behavior characteristics and elucidate the mode of male sperm transfer. Our research will help to explore the evolutionary mechanisms of insect genital structures.

## ﻿Material and methods

### ﻿Insect collection and rearing

The egg-infested wood (tree segments of oak trees *Quercusdentata* Thunb.) were cut down and stored in a temporary laboratory at Huabo Mountain in Kuandian County, Liaoning Province in northeastern China from 2020 to 2022. In the peak period of adult emergence of the second year (May to June and October to November of 2021–2023), the newly emerged adults were collected every day, and their emergence times were recorded. These adults were then individually placed in plastic boxes and promptly transferred to the Entomology Institute of Guangxi University (China) for separate rearing to sexual maturity. Then, twenty females or males of similar age were maintained separately in cages (40 × 30 × 30 cm) containing fresh chestnut branches (*Castaneamollissima* Blume) to simulate the habitat. A small amount of water was provided to maintain humidity in the cage. The rearing temperature was maintained at 25 ± 1 °C and humidity 75 ± 5% under a 14:10 h (L:D).

### ﻿Mating behavior observation

In the preliminary experiment, the adults were observed 24 h per day by people who took shifts for a week to check the circadian rhythms of mating activities. Then, five virgin females and five virgin males (7–10 days old) were randomly selected and placed in a transparent box to observe their mating. After the mating behavior of a pair of couples was recorded, they were removed to the new box immediately, and a new virgin male and female were added to the transparent box to maintain five virgin females and five virgin males. Their courtship behavior, mating process, copulation duration, and the activities of the mating-related structures were observed from noon to midnight. The mating processes were recorded using a Nikon D7100 digital camera. The duration of mounting, the duration of single expansion, and the duration of total expansion were recorded in detail. The duration of one single expansion of less than one minute was defined as a short expansion, and more than one minute was defined as a long expansion (Tong et al. 2024). At the end of the observation, males and females were separated and reared in different cages.

### ﻿Freeze-fixation of copulating pairs

The *M.diphysis* pairs in copula were frozen through carbon dioxide aerosol spray compressed in hydraulic cans and were immediately fixed in Carnoy’s solution at room temperature for 24–48 h to stabilize the interactions of their genital structures and preserved in 75% ethanol.

### ﻿Microscopic observations

The male and female genital systems of *M.diphysis* adults were dissected under a stereomicroscope (Nikon, SMZ800N). Photographs were taken with a Keyence VHX 6000 digital microscope. For scanning electron microscopy, the endophallus (internal sac) was dissected and then dried for 20 minutes on a glass slide. After that, the samples were coated with a film of gold in a sputter coater (Cressington, 108auto) and finally observed by the scanning electron microscope (FEI, Quattro S) at 5 kV.

### ﻿Statistical analyses

The length and width of the male and female genital structures were measured three times using Image-J ver. 1.8.0 (*N* = 5 each for males and females). Multi Timer ver. 2.12.1 was used to record the total duration of mating, the total duration of expansion, the duration of a single long expansion, and the duration of a single short expansion for each pair of beetles (a total of 24 pairs were observed). The number of expansions during mating was also recorded. All the data were subjected to statistical analysis using SPSS 20.0, and means and standard errors were calculated.

## ﻿Results

### ﻿Mating process

The mating behavior of *M.diphysis* mainly occurs in the afternoon. The complete mating process can be divided into three stages: meeting and mounting, expansion and ejaculation, and guarding after copulation. The detailed process of each stage is described as follows.

In the stage of meeting and mounting, the males exhibit a higher level of activity to search for a suitable mate. Upon approaching a female, the male mounts her back rapidly, and then the female shakes her body. Subsequently, the male flexes his abdomen, extending the lateral lobes to make contact with the female copulatory pore.

This process lasts for 29.31 ± 5.74 min (mean ± SE, *N* = 24) (Table 1), ranging from 9 s to 2 h. The male then extends the median lobe and expands the endophallus into the copulatory pore at the end of the female abdomen.

**Table 1.** Duration of the mating process of *Moechotypadiphysis* (mean ± SE, *N* = 24).

Mounting duration (min)	Short expansion duration (s)	Long expansion duration (min)	Total expansion duration (min)	Expansion times (min)	Total mating duration (min)
29.31 ± 5.74	3.85 ± 2.12	4.29 ± 0.35	6.56 ± 0.57	2.39 ± 0.27	70.81 ± 11.15

**Figure 1.**
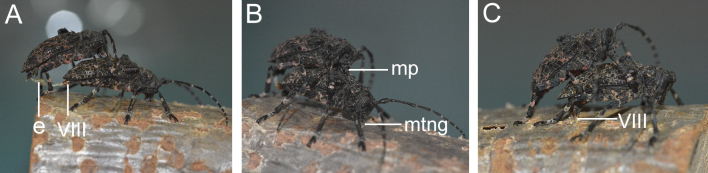
Mating process of *Moechotypadiphysis***A** male expands the endophallus into the female ovipositor **B** male uses the maxillary palps to lick the female back, and the female carves grooves on branch **C** after copulation, the female lays eggs. Abbreviations: e, endophallus; mp, maxillary palps; mtng, manufacture the notch grooves; VIII, the eighth abdominal segment.

In the stage of expansion and ejaculation (Fig. 1A), approximately 20% of males (5/24) expand only once; however, 80% of males (19/24) exhibit multiple expansions. The expansion can be divided into short and long expansions. The duration of short expansions ranges from 1.4 to 49 s, and the duration of long expansions ranges from 1.03 to 7.23 min. Among the males with multiple-expansions behaviors, about 15% of males (3/19) engage in multiple short expansions, and 85% of males (16/19) engage in multiple long expansions. Notably, the duration of the first expansion is usually the longest. During the process of endophallus expansion, the male rubs the base of the female elytra with his forelegs quickly, wiggles his antennae and touches the female with his maxillary palps (Fig. 1B). The average durations of a single expansion, total expansion and total mating for males are 2.39 ± 0.27 min, 6.56 ± 0.57 min, and 70.81 ± 11.15 min (mean ± SE, *N* = 24), respectively (Table 1).

After mating, the male pulls out the endophallus and mounts the female back to prevent other males from approaching the female (Fig. 1C). During the post-copulatory guarding stage, the female can carve grooves on the branch with her mandibles and lay eggs, completing the mating process.

### ﻿Genital structures of the two sexes

**Figure 2.**
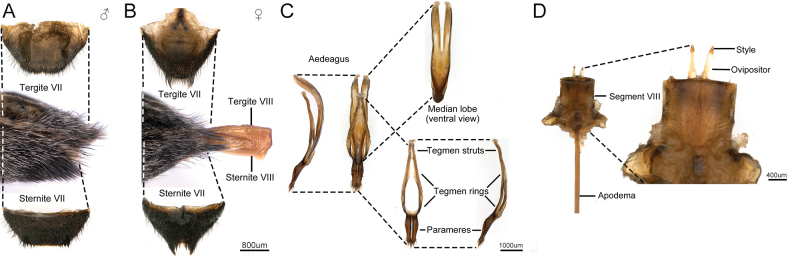
Abdominal apex and genital structures of the male and female *Moechotypadiphysis* adults **A** male **B** female **C** median lobe and tegmen **D** segment VIII and ovipositor (ventral view).

The male and female abdomen of *M.diphysis* adults differ on sternite VII and tergite VII. The male sternite VII and tergite VII are wider and straighter (Fig. 2A), while the female sternite VII and tergite VII are narrower and they have a noticeable indentation at the apex (Fig. 2B).

The aedeagus (Fig. 2C) is formed by the combination of the median lobe with the tegmen, which consists of the paired parameres (lateral lobes) connected to the tegminal struts by the tegminal ring. The tegmen is 432.74 ± 63.59 μm (mean ± SE, *N* = 5) long and 1036 ± 26.36 μm (mean ± SE, *N* = 5) wide, and the paramere is 1176.78 ± 37.06 μm (mean ± SE, *N* = 5) long and 227.20 ± 16.18 μm (mean ± SE, *N* = 5) wide. The parameres of *M.diphysis* are longer and straighter, compared with those of the cerambycidea *Psacotheahilaris* (Pascoe, 1871) and *Gleneacantor* (Fabricius, 1787) (Fukaya and Honda 1992; Lu 2007), and equipped with sensory hairs of different lengths (Fig. 2B). The median lobe is 4737.48 ± 310.15 μm (mean ± SE, *N* = 5) long and 937.25 ± 111.52 μm (mean ± SE, *N* = 5) wide, and is sclerotized and curved (Fig. 2C).

The female genitalia consist of segment VIII and the ovipositor enclosed within it (Fig. 2D). Segment VIII is trapezoidal, 1961.63 ± 113.90 μm (mean ± SE, *N* = 5) long and 1604.64 ± 128.83 μm (mean ± SE, *N* = 5) wide.

### ﻿Reproductive systems of both sexes and the internal coupling of genitalia

**Figure 3.**
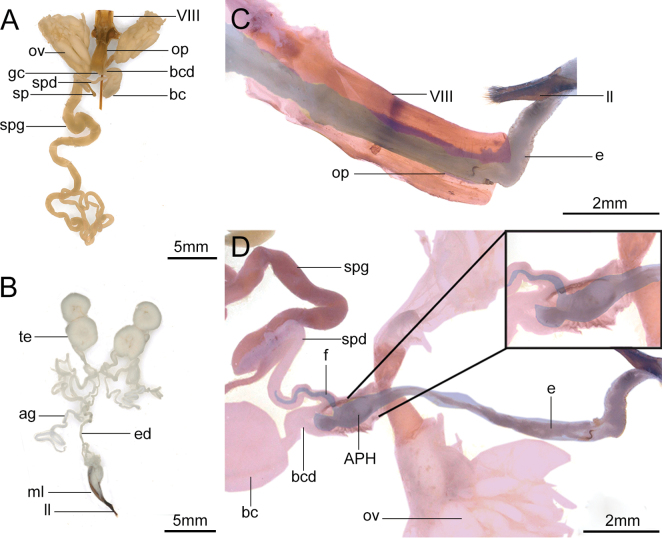
Reproductive system and the genitalia internal connection of *Moechotypadiphysis***A** reproductive system of the female **B** reproductive system of the male **C** endophallus expands into segment VIII, and the ovipositor **D** apical phallomere enters into the bursa copulatrix duct and flagellum enters into the spermathecal duct. Abbreviations: ag, accessory gland; APH, apical phallomere; bc, bursa copulatrix; bcd, bursa copulatrix duct; e, endophallus; ed, ejaculatory duct; f, flagellum; ll, lateral lobe; ml, median lobe; op, ovipositor; ov, ovary; sp, spermatheca; spd, spermathecal duct; spg, spermathecal gland; te, testis; VIII, the segment VIII.

The female reproductive system is composed of ovaries, lateral oviducts, middle oviduct, genital chamber, ovipositor, bursa copulatrix duct, bursa copulatrix, spermathecal duct, spermatheca, and spermathecal glands (Fig. 3A). The paired lateral oviducts of *M.diphysis* converge towards the middle to form a middle oviduct. The middle oviduct, the genital chamber and the ovipositor are three structures connected from top to bottom. There is also a bursa copulatrix duct at the junction of the middle oviduct and the genital chamber, and the bursa copulatrix duct is connected with the bursa copulatrix. The base part of the bursa copulatrix duct branched out into a spermathecal duct, which is successively connected with the spermatheca and the spermathecal glands. The bursa copulatrix is spherical-shaped, significantly enlarged, with a clearly curved base. The spermatheca is brown, rod-shaped and has 18 hardened rings that resemble springs (Fig. 3A).

The male reproductive system is composed of the testes, one pair of vasa deferentia, two pairs of accessory glands, and one ejaculatory duct (Fig. 3B). The two pairs of testes are white, well-developed and spherical-shaped. After each pair of testes are connected, they enter into the lateral vas deferens. Two pairs of accessory glands of similar length are fused at the base of the lateral vasa deferentia in *M.diphysis*. Two lateral vasa deferentia merge into the ejaculatory duct. The ejaculatory duct is connected to the endophallus. The endophallus is folded inside the middle lobe when unmated (Fig. 3B).

**Figure 4.**
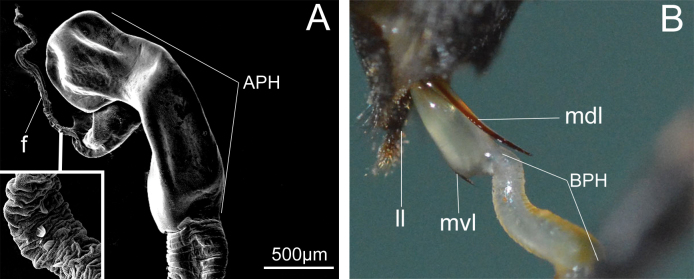
Morphology of a part of endophallus in *Moechotypadiphysis***A** flagellum and apical phallomere of the endophallus **B** the base of the endophallus is located between the median ventral leaf and median dorsal leaf. Abbreviations: APH, apical phallomere; BPH, basal phallomere; f, flagellum; ll, lateral lobes; mvl, median ventral leaf; mdl, median dorsal leaf.

Initially, the mechanical connection of male and female genitalia is established through the median ventral leaf and the median dorsal leaf of the median lobe (Fig. 4B). At the beginning of mating, the male touches the end of the female abdominal copulatory pore with the parameres and then expands the median ventral leaf of the median lobe into the female segment VII. When the male genitalia are fully coupled to the female genitalia, the endophallus is closely pressed against the inner wall of the female genitalia. Specifically, the endophallus is sequentially expanded into segment VIII, the ovipositor (Fig. 3C), the bursa copulatrix duct and the spermathecal duct (Fig. 3D). Finally, the apical phallomere of the endophallus is expanded into the bursa copulatrix duct to form a stable connection. The flagellum is located just at the opening of the spermathecal duct and enters the vicinity of the spermatheca by expanding the spermathecal duct (Fig. 3D).

### ﻿Male endophallus in mating

**Figure 5.**
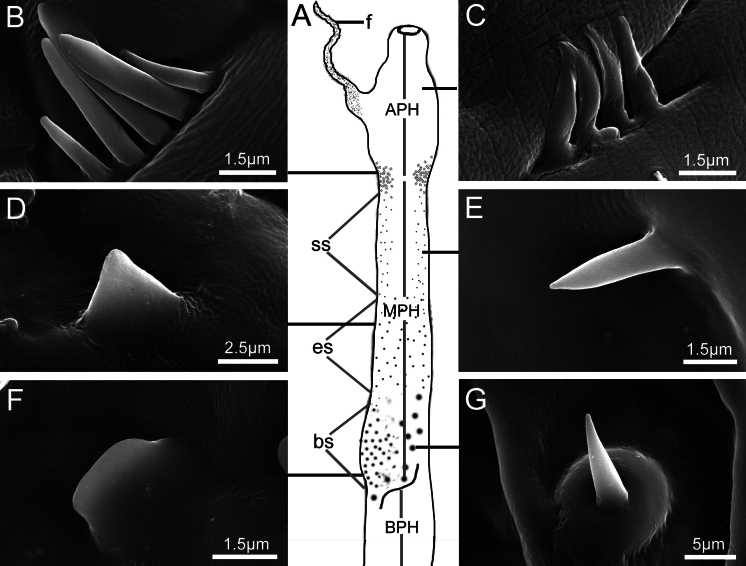
The diverse microstructures on the endophallus surface in *Moechotypadiphysis***A** surface of the endophallus showing the areas of apical and basal phallomere without spines, and the area of median phallomere with spines **B, C** apical phallomere covered with two different types of leaf-like microstructures **D** middle part of the median phallomere covered with short spines **E** upper part of the median phallomere covered with elongated spines **F** lower part of the median phallomere covered with broad spines **G** region of broad spines on the median phallomere surrounded by sensilla basiconica. Abbreviations: APH, apical phallomere; BPH, basal phallomere; bs, brosd spines; es, elongsted spines; f, flagellum; MPH, median phallomere; ss, short spines.

The endophallus is a membranous and tubular structure. Its apical region is composed of an apical phallomere and a flagellum (Fig. 4A). The endophallus is located inside the median lobe of the aedeagus when in repose. It is turned outward from the median lobe (Fig. 4B) and moves to the bursa copulatrix duct and the spermathecal duct during copulation. The endophallus is covered with various microstructures except for the flagellum (Fig. 5). The apical and the basal areas of the phallomere are without spines, while the median phallomere is the area with spines. There are elongated spines, short spines and broad spines in the upper, middle and lower parts of median phallomere (Fig. 5A).

If the median ventral leaf and median dorsal leaf of the median lobe are accidentally disconnected before complete eversion of the endophallus, the endophallus will fail to fully enter the genital chamber, leading to the separation of genitalia and the failure of mating. In such instances, the male needs to reinitiate the process. After the complete eversion of the endophallus, the microstructures of the endophallus contact different areas of the female genital tract.

The surface of the apical phallomere is covered with two different types of leaf-like microstructures (Fig. 5B, C), which are in close contact with the female bursa copulatrix duct. The short spines (Fig. 5D) and elongated spines (Fig. 5E) are in close contact with the female genital chamber. The broad spines (Fig. 5F) and a few sensilla basiconica (Fig. 5G) get in contact with the female ovipositor. However, none of the microstructures penetrate the female genital tissues.

### ﻿Clamping function and morphological characteristics of male leg

The legs serve as a crucial control structure in male longhorn beetles, facilitating their ability to grasp and secure the female during mating. The forelegs adeptly hold the base of the female elytra. The middle legs firmly secure the midsection of the female body. The hind legs typically provide support on two sides. No matter how the female shakes, the male can utilize the three pairs of legs to firmly grasp the female during mating.

**Figure 6.**
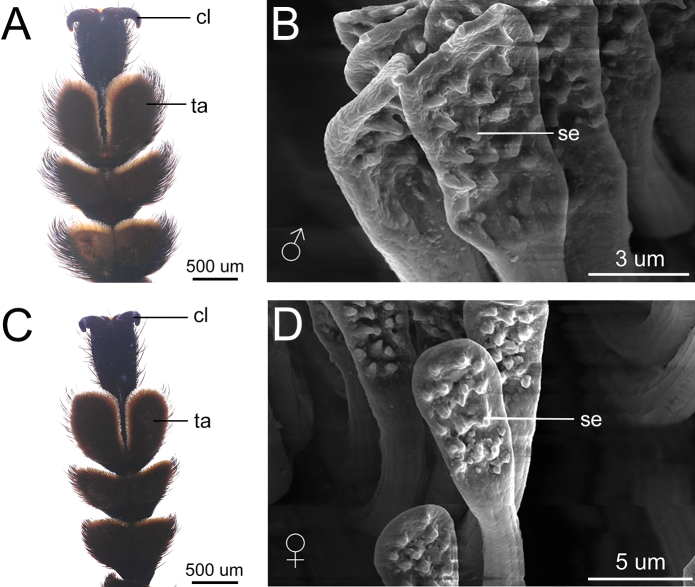
Protarsi and setae of male and female in *Moechotypadiphysis***A** male protarsus **B** male setae **C** female protarsus **D** female setae. Abbreviations: cl, claws; se, setule; ta, tarsus.

There is no difference in the structure of the protarsus of the *M.diphysis* between males and females. However, males and females have differences in the shape of the setae and the setules on the surface of the plates. The male protarsus is wider (Fig. 6A) than the female protarsus (Fig. 6C), the setae are spoon-like and the setules are blunter (Fig. 6D).

## ﻿Discussion

In this study, we investigated the copulation process, genital structures, and the freeze-fixated pairs in the copula of *M.diphysis*. There were multiple endophallus expansions (the membranous endophallus expands and enters into the female genital tract) of various durations during *M.diphysis* mating. The short expansion ranges from 1.4 to 49 s, and the long expansion ranges from 1.03 to 7.23 min. During copulation, male tarsi continuously grasped female elytra, thorax, and abdomen to help the male to initiate and maintain copulation. The apical phallomere of the endophallus are in contact with the female bursa copulatrix duct. The microspicules on the surface of the everted male endophallus directly contact the female genital tract. This appears to secure the connection between the male and female genitalia.

The mating processes of Cerambycidae are basically similar, however, there are differences in expansion times and copulation durations among different species (Kim et al. 2006; Fonseca and Zarbin 2009; Luo et al. 2011). For instance, most males of *Monochamusalternatus* (Hope, 1842) and *Psacotheahilaris* (Pascoe, 1871) perform multiple expansions in a single mating. Each expansion can transfer sperm. The copulation duration can range from seconds to minutes (Fukaya and Honda 1992; Kobayashi et al. 2003). In contrast, *Gleneacantor* (Fabricius, 1787) males expand only once during mating, and their copulation can last several hours (Li et al. 2015). The males of *M.diphysis* frequently exhibit multiple expansions. There are two possible reasons for their multiple expansions: First, it may be attributed to reproductive stress resulting from sperm competition, which has led males to evolve multiple strategies to counteract this stress (Cordero-Rivera 2017). We hypothesize that the multiple expansions could increase male fertilization success by enhancing sperm transfer, thereby potentially producing more offspring. Second, the extension of the male median ventral leaf appears to be unstable. Observations of the mating process reveal that the initial seconds of endophallus expansion coincide with the extension of the male median ventral leaf. The short expansion events are likely indicative of the failed mating, without or with only a small amount of sperm transferred. Therefore, the male needs multiple expansions in order to successfully transfer sperm. We need more experiments to test these hypotheses in the future.

Some structures of the male genital can control females during mating, for example, the grasping apparatus (Arnqvist and Rowe 2005; Edvardsson and Canal 2006; Maroni et al. 2023), achieving rapid fertilization (Eberhard and Huber 1998) or removing the sperm of the competitor (Córdoba-Aguilar 1999; Simmons 2001). In the bruchid beetle *Callosobruchusmaculatus* (Fabricius, 1775), the spines of the endophallus penetrate the bursa copulatrix (Crudgington and Siva-Jothy 2000; Blanckenhorn et al. 2002). In other species, such spines can reinforce the mechanical coupling of the female and male genitalia (Düngelhoef and Schmitt 2006; Rönn and Hotzy 2012; Shcherbakov 2023). The surface microstructures (such as spines) of the endophallus of Cerambycidae vary greatly in morphology (Kasatkin 2006; Lu et al. 2007; Hubweber and Schmitt 2010). In *Psacotheahilaris*, the spines are believed to function to remove sperm from competitors (Yokoi 1990). At present, we find that the spines may function to stabilize fertilization due to their contact with the female genital tract. This finding is similar to other research on longhorn beetles, including *Prionoplusreticularis* (White, 1843) (Edwards 1961) and *Dorysthenesgranulosus* (Thomson, 1861) (Tong et al. 2024). In addition, there are some sensilla basiconica on the endophallus of *M.diphysis*. These sensilla basiconica have a smooth surface without pores and a ring-shaped base. Based on their morphological characteristic, we believe that these sensilla basiconica are more likely to play a role in mechanical perception (Kerkut and Gilbert 1985; Keil 1997; Zhuge et al. 2010; Zhou et al. 2015). They may serve to locate the female genital tract or receive stimulation from the genital tract.

Besides controlling females, the evolution of the male genitalia may also be related to female oviposition. For example, the males of CerambycidaeTrachyderini or Torneutini have a short endophallus that matches the smaller ovipositor in females (Kasatkin 2006). Compared with other longhorn beetles of genera *Echinovelleda* Breuning, 1936, *Meges* Pascoe, 1866 and *Pseudomeges* Breuning, 1944 (Bi et al. 2022, 2024), the endophallus of *M.diphysis* is significantly different. The flagellum of *M.diphysis* is located on the side of apical phallomere, which is physically more compatible with the spermathecal duct of a female. In *M.diphysis*, the shape of the endophallus of the male and the ovipositor of the female are identical. Therefore, our results support the idea that the morphology of the endophallus of the male and the ovipositor of the female have evolved in mutual adaptation.

The female reproductive fluid (FRF) can generate paternity biases by affecting key traits in sperm competition (Higginson et al. 2012; Pinzoni et al. 2024). The secretions from the spermathecal glands of the honeybee *Apismellifera* (Linnaeus, 1758) and the cotton boll weevil *Anthonomusgrandis* (Boheman, 1843) have been shown to contribute to the activation and sustained movement of sperm (Koeniger 1970; Ruttner and Koeniger 1971; Villavaso 1975). In *M.diphysis*, the sperm is transported to the spermatheca through the flagellum of the endophallus. The spermatheca is connected to the well-developed spermathecal glands. The spermathecal glands of *M.diphysis* are significantly more developed than those of *Monochamusalternatus* (Hope, 1842) and *P.hilaris*. Therefore, we hypothesize that the *M.diphysis* spermatheca may participate in improving sperm vitality or selecting sperm by some secretions of the spermathecal glands.

Generally, the protarsus of male insects is used to grasp the female (Bergsten et al. 2001; Bergsten and Miller 2007; Green et al. 2013), and the setae on the protarsus have the effect of increasing adhesion (Zhang and Liang 2012; Xiong et al. 2014). The morphological structure of the setae in the protarsus of *M.diphysis* males is similar to that of the *Philonthuscognatus* (Stephens, 1832) males. The most unusual feature of these setae both in *M.diphysis* and *P.cognatus* is the presence of setules on the surface of the plates. Stork (1980) proposed that these structures may act as antimatting devices. Therefore, we believe that the setae of the *M.diphysis* belongs to a kind of adherent seta, and males have a wider protarsus, and their greater number of setae may be better for holding the female.

Our study has unveiled the mating behavior and how males use their genital structures for sperm transfer in *M.diphysis*. We also found that males have a multiple-expansions mating pattern, and we suspect that this reproductive strategy is aimed at reducing the difficulty of sperm competition. Finally, we briefly discuss the possible reason why the spermathecal glands of the *M.diphysis* female are so much better developed than other longhorns with multiple expansion patterns. This is likely related to female choice. It will be necessary to add more cerambycid beetles to increase the rigor and quality of similar studies of insect genitalia and utilize some of the newer methods of investigation, such as laser ablation, X-ray cineradiography, and micro-CT, to investigate the function of the internal and external genitalia in more detail (Schmitt and Uhl 2015).

## ﻿Additional information

### Author ORCIDs

Xin Tong https://orcid.org/0000-0003-1926-0024

### Data availability

The data supporting the findings of this study are available from the corresponding author upon reasonable request.
